# Open Repair of Large Hepatic Artery Pseudoaneurysm Without Collateral Circulation: A Case Report

**DOI:** 10.3389/fsurg.2022.791553

**Published:** 2022-03-30

**Authors:** Xin Wen, Xiyang Chen, Jichun Zhao, Xin Luo, Qiang Guo, Xiaojiong Du, Ding Yuan, Bin Huang

**Affiliations:** Department of Vascular Surgery, West China Hospital, Sichuan University, Chengdu, China

**Keywords:** hepatic artery pseudoaneurysm, open surgery repair, endovascular approach, reconstruction, good prognosis

## Abstract

Hepatic artery pseudoaneurysm is a rare arterial disease. This case report describes a patient with hepatic artery pseudoaneurysm who presented with recurrent epigastric pain over a 4-month period. Computed tomography angiography (CTA) showed aneurysmal enlargement of the hepatic artery measuring 55 mm × 46 mm. The angiographic information is as follows: (1) the common hepatic artery originated from the superior mesenteric artery; (2) the proper hepatic artery originated from the common hepatic artery; (3) the proper hepatic aneurysmal disease had no collateral circulation. After careful consideration, the patient underwent an open surgical repair (OSR). The patient recovered well without any associated complications. The 1-year follow-up of patients did not reveal any relevant complications. The treatment choice, puzzles, and reflections of this case are all discussed in this article.

## Introduction

Hepatic artery aneurysm (HAA) is a rare aneurysm with an overall prevalence of 0.002–0.4%, accounting for about 20% of visceral aneurysms and a rupture rate of 44% (1, 2). Arterial pseudoaneurysms account for 25–80% of reported cases and usually occur after medically induced injury or penetrating or blunt liver injury, resulting in symptomatic presentation of these aneurysms (3–5). HAA is the second most common visceral aneurysm after splenic artery aneurysm (6–10). Most common cases of HAA were observed during the sixth decade of life, with a 3:2 male predominance (1, 4). Majority of HAA cases are extrahepatic (75–80%) (1, 11, 12). A solitary aneurysm with multiple HAAs was reported in only 8% of cases (1). The risk factors of HAA include fibromuscular dysplasia, bacterial endocarditis, vasculitis, systemic lupus erythematosus or polyarteritis nodosa, Takayasu arteritis, Wegener granulomatosis, and congenital causes such as Marfan syndrome, Ehlers–Danlos syndrome, and Osler–Weber–Rendu syndrome (11–17). Abbas et al. (1) reported that patients with fibromuscular dysplasia and polyarteritis nodosa are at significant risk of HAA rupture, accounting for 50% of ruptured HAA. Hepatic artery pseudoaneurysm often occurs after iatrogenic injury or penetrating or blunt liver trauma, leading to symptomatic presentation of these aneurysms (3–5). The onset, diagnosis, treatment options, and advantages and disadvantages of various options for this patient, prognosis of this disease, and reflections of this case are discussed in this article.

## Case Report

**Figure 1:**
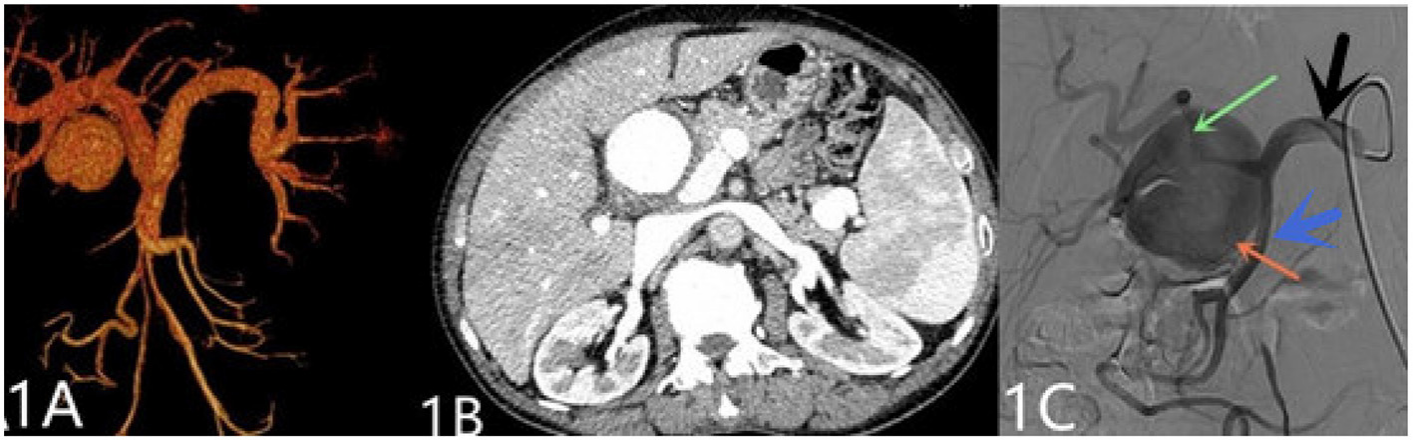
**(A)** Shows the pre-operative three-dimensional CT imaging of the hepatic pseudoaneurysm. **(B)** Displays the hepatic pseudoaneurysm shown by CT in the pre-operative patient, with a maximum cross-section of about 55 mm × 46 mm. **(C)** Shows the hepatic pseudoaneurysm shown by hepatic arteriography; the red arrow indicates the hepatic artery pseudoaneurysm; the green arrow indicates the proper hepatic artery; the black arrow points to the common hepatic artery; and the blue arrow points to the gastroduodenal artery.

**Figure 2:**
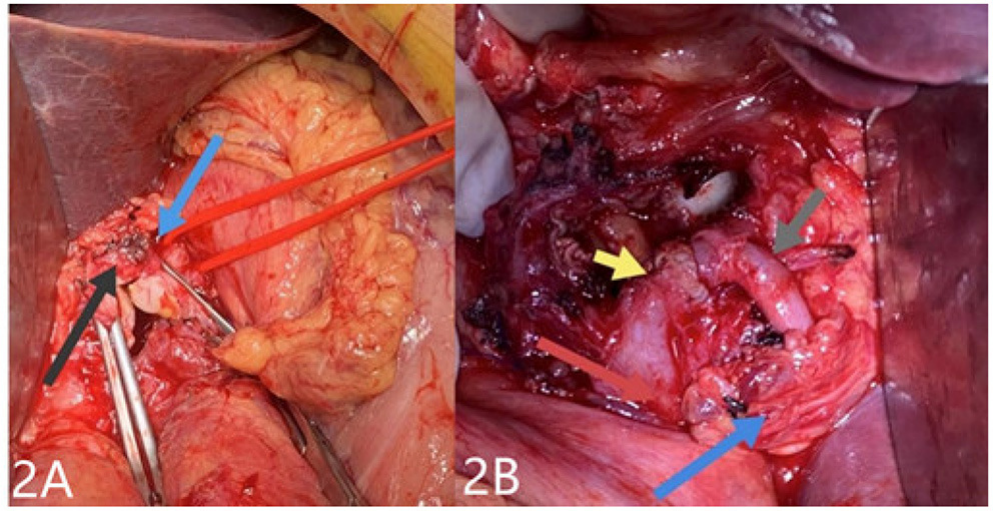
After resection of the hepatic pseudoaneurysm, in **(A)**, the blue arrow points to the proximal proper hepatic aneurysm while the black arrow points to the distal after separation. In **(B)**, after end-to-end anastomosis was performed, the gray arrow points to the proximal end of the suture, the yellow points arrow to the distal, the navy-blue arrow points to the removed aneurysm wall, and the red arrow points to the duodenum.

**Figure 3:**
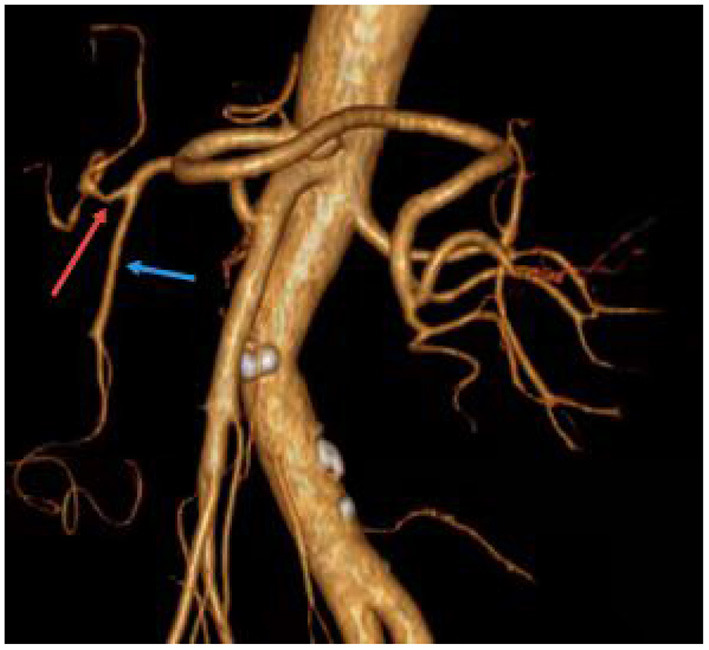
One year after operation, the patient showed good anastomosis of the proper hepatic artery. The red arrow indicates the reconstructed proper hepatic artery. The blue arrow points to the gastroduodenal artery.

A 69-year-old man was admitted to our hospital with complaints of intermittent epigastric pain for 4 months. Physical examination showed slight pressure pain in the upper abdomen but no rebound pain, and he had blood pressure of 136/84 mmHg, pulse of 96/min, and temperature of 36.3°C. Laboratory values on admission were as follows: 11.4 μmol/L direct bilirubin (DBIL), 237 IU/L alanine aminotransferase (ALT), 120 IU/L aspartate aminotransferase (AST), 106 g/L hemoglobin, 819 × 10^9^/L platelets, 5.11 × 10^9^/L white blood cells, and negative results of other blood tests. CTA revealed a 46 mm × 55 mm proper hepatic artery aneurysm arising from the common hepatic artery with multiple calcifications and local mural thrombosis (Figures 1A,B). Considering the risk of rupture, angiography was performed and endovascular treatment was prepared if needed. A 45 mm × 50 mm aneurysmal disease was observed about 30 mm from the initial segment of the proper hepatic artery without collateral vessels (Figure 1C). Deploying the stent was difficult considering the tortuosity of the delivery route. Otherwise, embolization was also excluded for the following reasons: first, the patient's hepatic artery pseudoaneurysm had no collateral vessels, which may lead to liver necrosis after embolization; second, the HAA was so large that it may compress the biliary tract and duodenum, causing jaundice, gastrointestinal obstruction, and even duodenum fistula. OSR was performed by right subcostal incision, approaching the proper hepatic artery pseudoaneurysm through the right gastrocolic ligament. The size of the pseudoaneurysm e was ~50 mm × 60 mm and closely adhesive to the omental sac and surrounding tissues. It is located between the medial duodenum, the head of the pancreas and the superficial part of the bile duct. Given the obvious peripheral adhesions, we did not explore the periphery of the aneurysm to reduce the possibility of tissue damage. The proximal and distal parts of the proper hepatic artery was mobilized, and the sac of the pseudoaneurysm was directly opened (Figure 2A). After removed the amount of thrombus from the sac, direct end-to-end anastomosis was performed with 6-0 Prolene, because the inflow and outflow vessels were adjacent (Figure 2B). The operation went smoothly. Hepatic artery clamp time was 25 min. Post-operatively, the patient had no special discomfort. Three days later, the patient was transferred to another hospital to continue his recovery without any complication. The patient's 1-year follow-up did not reveal any late complications (Figure 3). The results of blood reexamination showed 3 μmol/L DBIL, 30 IU/L ALT, and 34 IU/L AST.

## Discussion

In spite of their rarity with a reported incidence rate of 0.01–0.2%, visceral artery aneurysms hold a very important clinical significance, especially if we consider their natural history characterized by their propensity to rupture (18). These aneurysms are usually asymptomatic and difficult to detect until they rupture and cause abdominal pain and hypovolemic shock. The mortality rate after rupture of a visceral artery aneurysm remains quite high (30% as reported over the past decade) (19). The total prevalence rate of HAAs is 0.002–0.4%, accounting for about 20% of visceral aneurysms, and HAAs have a rupture rate of 44% (2, 20). HAAs are often related to hypertension, followed by malignancy and peripheral vascular disease. Other risk factors include chronic pancreatitis, trauma, and immune system disease (4).

HAAs can be diagnosed by ultrasound scan, computed tomography angiography (CTA), and digital subtraction angiography (DSA). According to *The Society for Vascular Surgery clinical practice guidelines on the management of visceral aneurysms*, CTA is the recommended diagnostic tool for patients who are thought to have HAA (Grade 1B); meanwhile, mesenteric angiography for pre-operative planning is recommended for patients with HAA who are considered for intervention (Grade 1B) (4).

Pseudoaneurysms can be distinguished from true aneurysms by antecedent clinical events such as iatrogenic injury or penetrating or blunt liver trauma along with specific imaging, focal arterial disruption in the setting of otherwise normal arteries, and inflammatory changes around an irregular aneurysm sac (4, 21). In contrast to true aneurysms, most pseudoaneurysms present with symptoms and are accompanied by gastrointestinal bleeding or bile bleeding (22, 23).

However, despite recent advances in therapeutic techniques and diagnostic tools, management of visceral artery aneurysm remains clinically challenging. Rupture is the most emergent and life-threatening situation for HAAs (4).

According to the clinical practice guidelines of the Society for Vascular Surgery on the management of visceral aneurysms, all hepatic artery pseudoaneurysms, given the high propensity of rupture and significant antecedent mortality, regardless of cause, should be repaired as soon as the diagnosis is made (Grade 1A) (4). However, in true HAAs, repair is recommended in the following situation: (1) all symptomatic HAAs regardless of size (Grade 1A); (2) asymptomatic patients without significant comorbidity, if true HAA is >2 cm (Grade 1A) or if aneurysm enlarges >0.5 cm/y (Grade 1C), and patients with significant comorbidities, if HAA is >5 cm (Grade 1B); (3) patients with vasculopathy or vasculitis, regardless of size (Grade 1C) and patients with HAA with positive blood cultures (Grade 1C) (4).

Treatment approaches mainly include the following: arterial embolization, endovascular stent–graft repair, or OSR (4). The ideal surgical procedure is to remove the aneurysm while maintaining hepatic circulation. An endovascular-first approach is recommended to all HAAs if it is anatomically feasible (i.e., if this approach maintains arterial circulation to the liver) (Grade 1A) (4). In patients with extrahepatic aneurysms, open and endovascular techniques are recommended to maintain liver circulation (Grade 1A) (4). Coil embolization of the affected artery is recommended for patients with intrahepatic aneurysms (Grade 1B). Meanwhile, resection of the involved lobe of the liver is recommended for patients with large intrahepatic HAA to avoid significant liver necrosis (Grade 1C) (4). OSR or endovascular repair of visceral artery aneurysms yields similar long-term results, but morbidity is significantly worse with open repair (24, 25). Therefore, endovascular techniques should be preferentially offered for anatomically suitable candidates. Overall, endovascular therapy has become the mainstream technique. However, open repair remains the therapeutic regimen with definite efficacy and is mostly chosen for HAA cases of ruptured, asymptomatic common hepatic artery (>2 cm), or asymptomatic common hepatic artery in patients with fibromuscular dysplasia or polyarteritis nodosa, and proper hepatic and proximal right or left hepatic branches (4, 5, 21, 24–26). A summary of treatment recommendations for extrahepatic aneurysms is shown in Table 1 (4).

**Table 1:** Summary of treatment recommendations for extrahepatic aneurysms.

**Location of extrahepatic HAA**	**Indication**	**Treatment**
Common hepatic artery	Ruptured	Open surgical ligation
	Symptomatic	Endovascular embolization
	Asymptomatic in patients with fibromuscular dysplasia or polyarteritis nodosa	Aneurysmorrhaphy
		Endovascular
		Covered stent
		Coil embolization
Proper hepatic	Same as above	Resection with arterial reconstruction Endovascular stent graft
Proximal right or left hepatic branches	Same as above	Resection with arterial reconstruction
		Endovascular stent graft

Although the endovascular-first approach to all HAAs is proposed if it is anatomically feasible (4), Young Erben et al. (27) reported that among HAA cases requiring intervention, 81% are treated by OSR (66.7% open reconstruction, 4.8% endoaneurysmorrhaphy alone, 4.8% patch and 4.8% ligation), and that 19% are treated with endovascular techniques (coil embolization performed 9.5% in the common and 9.5% in the right hepatic arteries). Furthermore, overall mortality was 14% (6% after elective OT, 40% for emergency OT, 0% for ET). Some retrospective case series have shown that the outcome for visceral artery aneurysms after OSR or endovascular repair yielded similar long-term results, but that morbidity is significantly worse with open repair than with the endovascular approach (25). Empirically, pseudoaneurysms tended to have higher mortality and reintervention rates (2). The main complications of endovascular treatment are hepatic ischemia, abscess, cholecystitis, possible recanalization, rebleeding, and internal fistula (27). In this case, we did not choose endovascular approaches mainly based on the following reasons: first, the patient's hepatic artery pseudoaneurysm with a huge size of about 45 mm × 50 mm had no collateral vessels. If we performed embolization, it is likely to compress the pancreas, duodenum, and bile duct, and increase the probability of pancreatitis, digestive tract obstruction, and jaundice, leading to liver necrosis. Second, the patient's hepatic artery pseudoaneurysm was too tortuous to place a covered stent. Mostly, OSR needs vascular grafts, including artificial blood vessels and great saphenous vein, to repair HAAs (4). However, we performed end-to-end anastomosis because of the short distance of the proximal to the distal part of the HAA.

## Conclusion

Although endovascular therapy is the first choice of treatment in most cases, open surgery still has its unique role. We should not only strictly grasp the indications of various surgical procedures but also make clinical decisions according to specific conditions of patients. What is more, timely and correct diagnosis is the first step in treatment; although an HAA is a rare disease, it can be potentially lethal especially if left untreated or becomes late diagnosed.

## Data Availability Statement

The raw data supporting the conclusions of this article will be made available by the authors, without undue reservation.

## Ethics Statement

Written informed consent was obtained from the individual(s) for the publication of any potentially identifiable images or data included in this article.

## Author Contributions

XW and XC were contributed equally to this article and mainly responsible for the collection of data and article writing. JZ and BH were mainly responsible for treatment plan selection, surgical design, and implementation. QG, XL, and XD were mainly responsible for data collection and surgical assistance. All authors contributed to the article and approved the submitted version.

## Funding

This work was supported by the Sichuan University Huaxi Nursing Discipline Development Fund (No. HXHL19045).

## Conflict of Interest

The authors declare that the research was conducted in the absence of any commercial or financial relationships that could be construed as a potential conflict of interest.

## Publisher's Note

All claims expressed in this article are solely those of the authors and do not necessarily represent those of their affiliated organizations, or those of the publisher, the editors and the reviewers. Any product that may be evaluated in this article, or claim that may be made by its manufacturer, is not guaranteed or endorsed by the publisher.
